# Data on macrobenthic prey from an essential western gray whale feeding habitat, Sakhalin Island, Russia, 2001–2015

**DOI:** 10.1016/j.dib.2019.103968

**Published:** 2019-05-24

**Authors:** Arny L. Blanchard, Natalia Demchenko, Lise A.M. Aerts, Sergei Yazvenko, Victor Ivin, Ilya Shcherbakov, H. Rodger Melton

**Affiliations:** a Blanchard Ecological, North Pole, AK, USA; b National Scientific Center of Marine Biology FEB RAS, Russia; c LAMA Ecological, Dallas, TX, USA; d LGL Limited, Sydney, British Columbia, CA, USA; e L.S. Berg State Research Institute on Lake and River Fisheries, Saint Petersburg, Russia; f Far Eastern Federal University, Vladivostok, Russia; g ExxonMobil, Houston, TX, USA

## Abstract

Data in this article presents data (means and standard deviations) for prey biomass from essential feeding habitats for the endangered western gray whale. Prey include Actinopterygii (primarily the sand lance *Ammodytes hexapterus*), Amphipoda, Bivalvia, Cumacea, Isopoda, and Polychaeta. Total prey biomass (sum of the six prey groups) is also presented. Statistical analyses document spatial and temporal trends in prey biomass concentrations. Multivariate analyses using canonical correspondence analysis characterize relationships of potential drivers of community changes.

**Table:** Specifications table

Subject area	Biology, marine ecology
More specific subject area	Marine macrobenthic ecology; gray whale prey biomass, spatial and temporal variability of marine benthic communities
Type of data	Table, figure
How data was acquired	Oceanographic survey
Data format	Analyzed
Experimental factors	Animals were identified to class or higher in the laboratory.
Experimental features	Benthic animals collected during oceanographic surveys adjacent to Sakhalin Island, Russia, 2001–2015 in a western gray whale feeding area. Laboratory taxonomic analyses focused on biomass of whale prey and classes were identified. Descriptive statistics summarizing the long-term database were determined and ANCOVAs conducted for the primary prey.
Data source location	Sakhalin Island, Russia
Data accessibility	Average biomass concentrations presented within this article.
Related research article	Blanchard, A.L., Demchenko, N.L., Aerts, L.A.M., Yazvenko, S.B., Ivin, V.V., Shcherbakov, I., Melton, H.R., 2019. Prey biomass dynamics in gray whale feeding areas adjacent to northeastern Sakhalin (the Sea of Okhotsk), Russia, 2001–2015. Mar. Environ. Res. 145, 123–136. https://doi.org/10.1016/j.marenvres.2019.02.008

**Table:** 

**Value of the data** • Data summarize benthic prey biomass concentrations from essential habitat for the endangered western gray whale. • Temporal variations in prey biomass concentrations are large and a significant source of variability in prey biomass. Amphipoda biomass, the primary prey for gray whales, demonstrated significant declines over time. • Understanding prey dynamics contributes to a better understanding of sources for change in gray whale populations, particularly for the endangered western gray whale. • The Sea of Okhotsk is undergoing significant climate-related ecosystem changes and the macrobenthic data provide biomass concentrations that support a more complete understanding of the extent of climatic influences.

## 1 Data

**Fig. 1:**
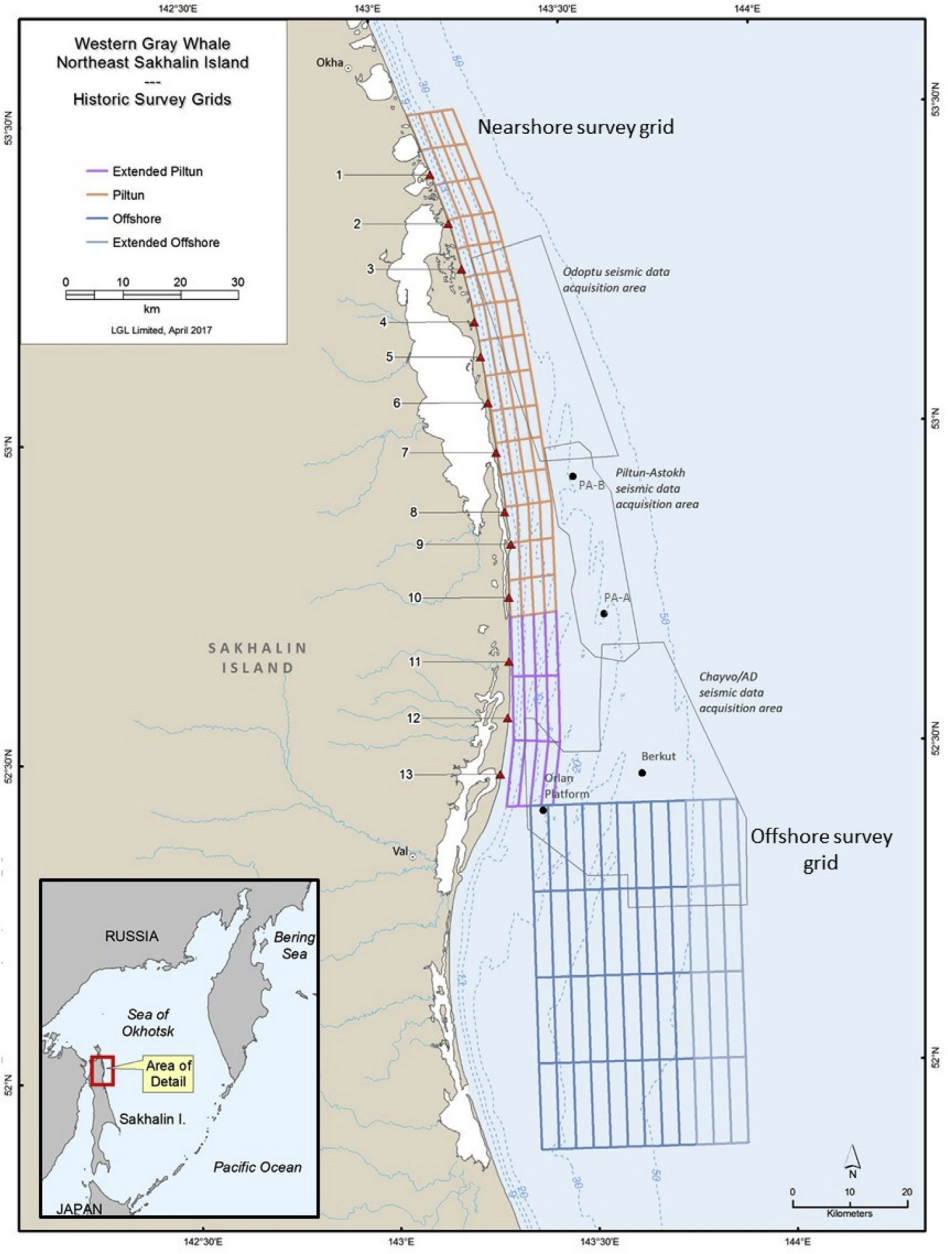
Sakhalin Island survey grids. Benthic sampling locations were either randomly selected within the grid or repeated from previous years. One location was selected for sampling in each grid and three replicate van Veen grabs were collected at each sampling location.

**Fig. 2:**
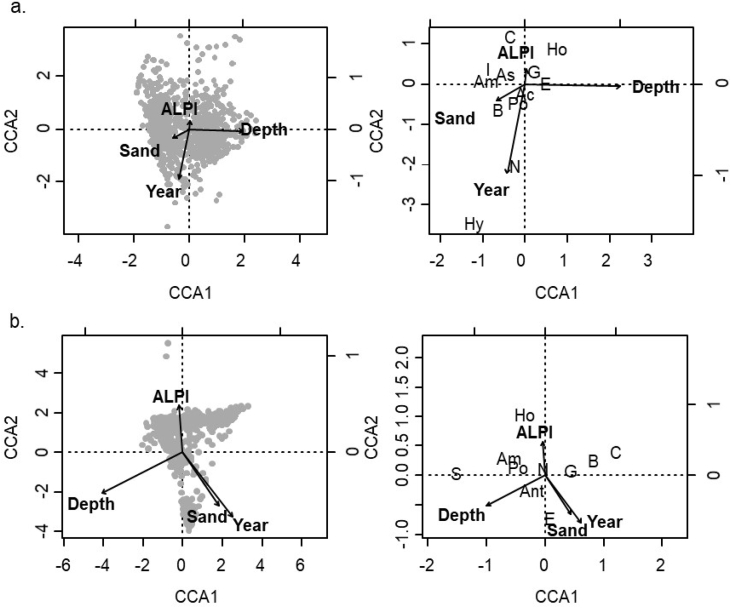
Canonical correspondence analysis (CCA) of the nearshore (a) and offshore (b) study areas, 2002–2015. The survey from 2001 was not included due to missing data. Plots on the left present the ordination by stations and the plots on the right side present species ordinations. The correlations of predictor variables are presented as biplots where the length and direction of an arrow represents the direction and strength of association with the axes. The positioning of an arrow in the direction of the spread of stations and location of a group label indicates joint associations. The faunal groups in the analysis are Am = Amphipoda, Ant = Anthozoa, As = Ascidiacea, B = Bivalvia, C = Cumacea, E = Echinoidea, G = Gastropoda, Ho = Holothuirodea, Hy = Hydrozoa, I = Isopoda, N = Nemertea, Pi = Pisces, and Po = Polychaeta. ALPI = the Aleutian Low Pressure Index.

Macrobenthic community biomass was determined from the northeastern Sakhalin Island gray whale feeding area, Sea of Okhotsk, Russia [1]. Bottom samples were collected from 2001 to 2015 to determine prey biomass characteristics and distributions. Biomass data presented here include 6 prey (Actinopterygii (primarily sand lance *Ammodytes hexapterus*), Amphipoda, Bivalvia, Cumacea, Isopoda, and Polychaeta), and total prey (sum of the six prey categories). Average biomass, sample size, and standard deviations are presented for the nearshore (2001–2015) and offshore (2001–2015) study areas (Fig. 1; Table 1, Table 2). Biomass values are also presented for feeding points (opportunistically sampled locations where whales were observed feeding) to determine if feeding areas had different biomass characteristics than other stations (Table 3). ANCOVA and Tukey multiple comparisons document long-term differences for benthic prey among years for the nearshore and offshore surveys (Table 4, Table 5, Table 6). Canonical correspondence analyses were conducted to investigate relationships between environmental predictors and community biomass structure using all macrofaunal groups (Fig. 2; Table 7) (see: Table 8).

**Table 1:** Sample size, averages, and standard deviations (SD) for the nearshore study area for Amphipoda, Bivalvia, Cumacea, Isopoda, Actinopterygii, Polychaeta, and the total prey (T6: sum of 6 prey) biomass, 2001–2015.

Year	N	Average Amphipoda	SD	Average Bivalvia	SD	Average Cumacea	SD	Average Isopoda	SD
2001	60	36.6	62.3	106.9	141.4	20.8	30.6	25.7	39.7
2002	88	32.2	58.7	27.6	56.1	12.5	29.8	13.8	20.5
2003	55	56.3	62.5	45.1	92.9	2.2	6.7	21.3	30.7
2004	45	46.8	50.5	21.9	28.7	0.8	2.4	10.2	13.4
2005	48	30.5	34.3	24.8	29.5	1.0	1.9	5.4	6.6
2006	60	28.2	33.5	15.2	38.1	2.7	8.2	11.1	11.1
2007	78	33.4	39.7	53.3	92.4	4.2	14.1	12.8	22.0
2008	67	37.2	32.4	35.5	47.2	1.8	4.9	3.9	3.1
2009	74	18.6	26.1	23.3	35.1	0.4	0.8	3.8	5.9
2010	79	29.4	37.7	25.1	35.7	5.1	42.7	7.0	9.1
2011	60	20.6	45.8	92.4	139.0	0.1	0.7	6.8	14.0
2012	73	38.4	44.9	56.2	70.9	0.3	0.7	10.2	13.9
2013_1	42	40.2	35.2	111.2	128.8	2.2	3.4	12.7	13.2
2013_2	70	16.8	21.6	58.2	83.3	0.5	1.1	8.0	10.2
2014	72	18.0	24.1	45.1	81.7	0.3	0.5	6.7	12.4
2015	63	19.8	28.5	55.3	94.3	0.4	0.6	4.4	5.5

Year	N	Average Actinopterygii	SD	Average Polychaeta	SD	T6	SD
2001	60	4.3	10.8	24.2	37.6	218.4	180.6
2002	88	6.2	10.1	8.6	8.5	100.8	98.9
2003	55	4.5	12.5	12.6	26.8	142.3	106.9
2004	45	49.4	88.2	6.5	7.2	135.5	113.6
2005	48	56.5	102.1	9.2	15.0	127.3	119.5
2006	60	13.7	32.0	3.2	6.6	74.0	60.3
2007	78	9.1	24.2	4.6	4.9	117.5	104.7
2008	67	3.0	6.8	5.2	6.2	86.4	55.1
2009	74	8.1	18.8	2.4	2.7	56.6	47.6
2010	79	12.7	30.2	4.1	5.9	83.3	65.3
2011	60	12.9	26.2	10.7	14.4	143.5	148.3
2012	73	3.6	14.3	6.6	7.2	115.1	87.6
2013_1	42	4.7	6.6	7.3	5.1	178.2	114.4
2013_2	70	6.1	10.4	10.7	16.2	100.1	84.3
2014	72	4.2	10.4	8.8	22.9	82.4	97.2
2015	63	23.0	37.0	7.7	15.0	110.7	101.2

**Table 2:** Sample size, averages, and standard deviations (SD) for the offshore study area for Amphipoda, Bivalvia, Cumacea, Isopoda, Actinopterygii, Polychaeta, and the total prey (T6: sum of 6 prey) biomass, 2001–2015.

Year	N	Average Amphipoda	SD	Average Bivalvia	SD	Average Cumacea	SD	Average Isopoda	SD
2002	36	268.5	313.4	50.0	79.9	24.7	30.9	0.0	0.0
2003	36	233.2	261.7	70.3	66.6	57.0	93.5	0.0	0.0
2004	32	246.7	213.3	116.0	110.6	19.7	29.7	0.0	0.0
2005	48	200.3	219.4	76.8	85.3	44.3	49.8	0.0	0.0
2006	48	184.9	191.9	46.1	146.7	10.8	12.1	0.0	0.0
2007	48	173.6	236.5	14.1	12.2	20.2	24.8	0.0	0.0
2008	48	170.0	190.2	12.7	11.3	2.9	6.5	0.0	0.0
2009	48	140.0	191.8	83.4	141.3	33.1	42.5	0.0	0.0
2010	48	169.0	194.2	68.3	72.9	49.8	60.4	0.0	0.0
2012	48	143.2	276.8	73.6	65.6	98.4	150.3	0.9	4.1
2013	48	119.7	143.0	179.7	332.0	42.4	60.5	4.3	14.0
2014	48	173.5	275.7	76.1	60.9	76.2	115.4	2.3	11.6
2015	48	132.3	194.1	193.8	329.1	63.9	103.2	0.5	2.4

Year	N	Average Actinopterygii	SD	Average Polychaeta	SD	T6	SD
2002	36	0.0	0.0	16.0	30.4	359.1	343.4
2003	36	0.0	0.0	17.1	15.0	377.5	264.7
2004	32	0.0	0.0	40.6	129.1	423.0	281.9
2005	48	0.0	0.0	20.7	25.3	342.0	250.1
2006	48	0.0	0.0	19.7	24.9	261.6	237.5
2007	48	0.0	0.0	15.5	14.2	223.4	235.0
2008	48	0.0	0.0	8.6	9.4	194.3	193.8
2009	48	0.0	0.0	13.3	17.6	269.8	213.3
2010	48	0.0	0.0	17.9	24.3	305.0	206.8
2012	48	0.1	0.4	21.4	24.2	337.7	315.4
2013	48	0.9	2.4	25.8	29.8	372.9	360.2
2014	48	1.5	5.7	24.0	26.8	353.5	295.5
2015	48	0.1	0.6	24.4	23.2	415.0	389.0

**Table 3:** Average biomass (g m^−2^) of six prey groups and total prey biomass from feeding points in the nearshore and offshore surveys 2002–2015.

Region	Year	Amp	Biv	Cu	Iso	Act	Poly	T 6
Nearshore	2002	72.9	13.9	1.2	16.1	4.0	3.0	111.1
	2003	83.5	19.3	2.5	30.1	41.1	4.0	180.5
	2004	44.3	17.7	0.8	14.9	39.6	2.4	119.7
	2005	63.8	30.9	3.2	8.5	51.3	7.8	165.5
	2006	41.2	17.0	0.8	9.7	4.3	3.9	76.8
	2007	53.5	47.9	0.9	8.2	16.5	4.8	131.7
	2008	51.8	28.5	1.6	10.6	0.4	3.3	96.1
	2009	32.8	20.5	1.6	18.3	34.6	4.4	112.3
	2010	72.4	47.7	5.0	14.2	48.8	9.1	197.2
	2011	112.9	11.1	1.7	23.9	0.0	6.9	156.4
	2012	67.1	32.1	2.5	13.0	0.4	9.8	124.9
	2015	32.4	51.8	0.5	10	8.3	3.9	106.8
Offshore	2002	264.9	35.8	16.0	0.0	0.1	8.0	324.7
	2003	379.5	48.9	9.4	0.0	0.0	3.6	441.4
	2004	192.4	57.4	35.7	0.0	0.0	23.4	309.0
	2005	65.7	25.7	15.8	7.1	1.3	5.0	120.6
	2006	201.0	37.8	96.8	0.0	0.0	25.4	361.0
	2007	449.2	61.7	15.8	0.1	0.0	37.1	563.9
	2008	174.1	32.3	18.7	0.0	0.0	15.1	240.2
	2009	274.8	26.3	3.9	0.0	0.0	8.4	313.3
	2010	296.4	28.8	22.9	0.0	0.4	18.7	367.1
	2012	472.7	44.7	1.7	0.0	0.0	15.7	534.8
	2015	695.2	162.3	0.0	0.1	0.0	72.4	930.1

Act = average Actinopterygii biomass, Amp = Amphipoda, Biv = Bivalvia, Cu = Cumacea, Iso = Isopoda, Poly = Polychaeta, and T6 = total biomass of 6-prey group. Feeding points were not sampled in every year within each study area.

**Table 4:** Tukey multiple comparisons among years for faunal groups for nearshore surveys, 2001–2015.

Comparison	Amphipoda	Bivalvia	Cumacea	Isopoda	Actinopterygii	Polychaeta	T6
Model	D + Y	Y	D + Y	D + Y	Y	D + Y	D + Y
TS Corr.	AR(3)	AR(3)	AR(2)	AR(3)	ARMA	ARMA	ARMA
2001–2002	0.9973	**<0.0001**	**<0.0001**	0.6426	1.0000	**<0.0001**	0.0647
2001–2003	0.8068	**<0.0001**	**<0.0001**	0.3322	1.0000	**<0.0001**	0.2530
2001–2004	**0.0006**	**<0.0001**	**<0.0001**	**0.0186**	0.0796	**<0.0001**	0.4522
2001–2005	1.0000	**<0.0001**	**<0.0001**	**<0.0001**	0.0782	**<0.0001**	**0.0331**
2001–2006	0.9977	**<0.0001**	**<0.0001**	0.0659	1.0000	**<0.0001**	**<0.0001**
2001–2007	0.9977	**0.0001**	**<0.0001**	**0.0079**	1.0000	**<0.0001**	**0.0031**
2001–2008	0.3991	**<0.0001**	**<0.0001**	**<0.0001**	0.9954	**<0.0001**	**0.0001**
2001–2009	0.2778	**<0.0001**	**<0.0001**	**<0.0001**	1.0000	**<0.0001**	**<0.0001**
2001–2010	0.9999	**<0.0001**	**<0.0001**	**<0.0001**	0.9991	**<0.0001**	**<0.0001**
2001–2011	0.5761	0.1673	**<0.0001**	**<0.0001**	0.9779	**0.0002**	0.0535
2001–2012	1.0000	**0.0049**	**<0.0001**	**0.0057**	0.8716	**<0.0001**	**<0.0001**
2001–2013	0.0556	0.1513	**<0.0001**	**<0.0001**	1.0000	**0.0076**	**0.0005**
2001–2014	**0.0325**	**0.0001**	**<0.0001**	**<0.0001**	1.0000	**<0.0001**	**<0.0001**
2001–2015	0.4273	**0.0096**	**<0.0001**	**<0.0001**	0.5685	**<0.0001**	**<0.0001**
2002–2003	**<0.0001**	1.0000	**0.0098**	1.0000	0.9888	0.9711	1.0000
2002–2004	**<0.0001**	1.0000	**<0.0001**	0.7780	**0.0200**	0.8720	0.9999
2002–2005	0.3116	1.0000	**<0.0001**	**0.0004**	**0.0247**	0.7152	1.0000
2002–2006	**0.0422**	**0.0207**	**<0.0001**	0.9830	0.9909	**<0.0001**	**0.0007**
2002–2007	**0.0454**	0.9595	**<0.0001**	0.6299	0.9739	0.7169	0.9991
2002–2008	**<0.0001**	0.9994	**0.0005**	**<0.0001**	0.6249	0.5288	0.7979
2002–2009	0.6574	0.9838	**<0.0001**	**<0.0001**	1.0000	**<0.0001**	**<0.0001**
2002–2010	1.0000	1.0000	**<0.0001**	**0.0102**	1.0000	**0.0023**	**0.0002**
2002–2011	0.9597	**0.0311**	**<0.0001**	**0.0001**	0.9935	0.9558	1.0000
2002–2012	0.8507	0.3228	**<0.0001**	0.5538	0.1436	0.8937	0.3501
2002–2013	0.1300	**0.0053**	**<0.0001**	**0.0005**	1.0000	0.0677	0.9652
2002–2014	0.0748	0.9616	**<0.0001**	**<0.0001**	0.9962	1.0000	**0.0003**
2002–2015	0.9757	**0.0052**	**<0.0001**	**<0.0001**	0.4592	1.0000	0.3689
2003–2004	**0.0079**	0.9995	**0.0003**	0.9708	**<0.0001**	1.0000	1.0000
2003–2005	0.9382	1.0000	**0.0232**	**0.0051**	**0.0001**	1.0000	0.9992
2003–2006	0.9979	**0.0030**	0.2291	1.0000	1.0000	**<0.0001**	**<0.0001**

Comparison	Amphipoda	Bivalvia	Cumacea	Isopoda	Actinopterygii	Polychaeta	T6
2003–2007	0.9981	0.9990	0.2593	0.9548	1.0000	1.0000	0.9295
2003–2008	1.0000	1.0000	0.9998	**0.0011**	0.9997	0.9998	0.3635
2003–2009	**<0.0001**	0.8494	**<0.0001**	**<0.0001**	0.9995	**0.0002**	**<0.0001**
2003–2010	**0.0055**	1.0000	**<0.0001**	0.0924	0.7974	0.2746	**<0.0001**
2003–2011	**<0.0001**	0.1325	**<0.0001**	**0.0019**	0.5232	0.2222	0.9999
2003–2012	0.3421	0.6998	**<0.0001**	0.9267	0.9154	1.0000	0.0857
2003–2013	**<0.0001**	**0.0370**	**<0.0001**	**0.0088**	0.9438	**0.0004**	0.6865
2003–2014	**<0.0001**	0.9992	**<0.0001**	**0.0002**	1.0000	0.9631	**<0.0001**
2003–2015	**<0.0001**	**0.0478**	0.2761	**<0.0001**	**0.0096**	0.9875	0.0612
2004–2005	**<0.0001**	1.0000	0.9998	0.3596	1.0000	1.0000	0.9884
2004–2006	**0.0001**	0.1689	0.8772	1.0000	**0.0001**	**0.0017**	**<0.0001**
2004–2007	**0.0002**	0.8120	0.8806	1.0000	**0.0001**	1.0000	0.8579
2004–2008	0.2101	0.9822	**0.0304**	0.3138	**<0.0001**	1.0000	0.2874
2004–2009	**<0.0001**	0.9999	0.9998	0.0630	**0.0175**	**0.0058**	**<0.0001**
2004–2010	**<0.0001**	1.0000	0.9978	0.9671	0.2546	0.7314	**<0.0001**
2004–2011	**<0.0001**	**0.0135**	0.9793	0.2820	0.8117	0.0989	0.9993
2004–2012	**<0.0001**	0.1641	0.9945	1.0000	**<0.0001**	1.0000	0.0688
2004–2013	**<0.0001**	**0.0022**	0.9999	0.6484	0.1257	**0.0001**	0.5926
2004–2014	**<0.0001**	0.8189	1.0000	0.1599	**0.0003**	0.8028	**<0.0001**
2004–2015	**<0.0001**	**0.0023**	0.6855	**0.0227**	0.8597	0.8892	0.0706
2005–2006	1.0000	**0.0292**	0.9996	**0.0325**	**<0.0001**	**0.0011**	**0.0016**
2005–2007	1.0000	0.9123	0.9998	0.4193	**<0.0001**	1.0000	1.0000
2005–2008	0.4911	0.9970	0.3038	1.0000	**<0.0001**	1.0000	0.9758
2005–2009	**0.0002**	0.9984	0.8539	1.0000	**0.0153**	**0.0120**	**<0.0001**
2005–2010	0.6591	1.0000	0.7306	0.9997	0.2469	0.8635	**0.0040**
2005–2011	**0.0057**	**0.0263**	0.5496	1.0000	0.8139	**0.0423**	1.0000
2005–2012	0.9999	0.2697	0.6527	0.5109	**<0.0001**	1.0000	0.7532
2005–2013	**<0.0001**	**0.0048**	0.9071	1.0000	0.1203	**<0.0001**	0.9992
2005–2014	**<0.0001**	0.9237	0.9480	1.0000	**0.0002**	0.5981	**0.0060**
2005–2015	**0.0041**	**0.0053**	0.9960	0.9999	0.8587	0.7011	0.8434
2006–2007	1.0000	**<0.0001**	1.0000	0.9999	1.0000	**0.0001**	**0.0091**
2006–2008	0.7867	**0.0002**	0.8433	**0.0204**	0.9998	**0.0027**	0.4071
2006–2009	**<0.0001**	0.6534	0.1493	**0.0015**	0.9981	1.0000	0.0512
2006–2010	0.1794	0.0585	0.0860	0.4998	0.7059	0.6281	1.0000
2006–2011	**0.0002**	**<0.0001**	0.0505	**0.0278**	0.4307	**<0.0001**	**0.0201**
2006–2012	0.9743	**<0.0001**	0.0623	0.9999	0.9323	**0.0003**	0.9163
2006–2013	**<0.0001**	**<0.0001**	0.2199	0.1105	0.9033	**<0.0001**	0.2573
2006–2014	**<0.0001**	**<0.0001**	0.2913	**0.0067**	1.0000	**<0.0001**	1.0000
2006–2015	**0.0001**	**<0.0001**	1.0000	**0.0002**	**0.0039**	**<0.0001**	0.5281
2007–2008	0.6318	1.0000	0.7784	0.1261	0.9999	1.0000	0.9991
2007–2009	**<0.0001**	0.1165	0.1209	**0.0308**	0.9891	**0.0021**	**<0.0001**
2007–2010	0.1780	0.8465	0.0755	0.9557	0.5618	0.7200	**0.0134**

Comparison	Amphipoda		Bivalvia	Cumacea	Isopoda	Actinopterygii	Polychaeta		T6
2007–2011	**0.0002**	0.7334		**0.0444**	0.2147	0.3186	**0.0330**	1.0000	
2007–2012	0.9744	0.9991		0.0554	1.0000	0.9728	1.0000	0.9635	
2007–2013	**<0.0001**	0.4725		0.2020	0.5667	0.8197	**<0.0001**	1.0000	
2007–2014	**<0.0001**	1.0000		0.2684	0.0998	1.0000	0.5775	**0.0246**	
2007–2015	**0.0001**	0.6370		1.0000	**0.0069**	**0.0016**	0.6526	0.9896	
2008–2009	**<0.0001**	0.2568		**<0.0001**	1.0000	0.5457	**0.0015**	**<0.0001**	
2008–2010	**0.0001**	0.9896		**<0.0001**	0.9966	0.0965	0.8576	0.1830	
2008–2011	**<0.0001**	0.3591		**<0.0001**	1.0000	0.0532	**0.0136**	0.9714	
2008–2012	**0.0355**	0.9475		**<0.0001**	0.2883	1.0000	1.0000	1.0000	
2008–2013	**<0.0001**	0.1487		**0.0002**	1.0000	0.3023	**<0.0001**	1.0000	
2008–2014	**<0.0001**	1.0000		**0.0004**	1.0000	0.9995	0.3830	0.3131	
2008–2015	**<0.0001**	0.2083		0.9288	1.0000	**<0.0001**	0.4112	1.0000	
2009–2010	0.1235	0.9953		1.0000	0.7615	0.9987	0.6194	**0.0448**	
2009–2011	1.0000	**0.0001**		1.0000	1.0000	0.9712	**<0.0001**	**<0.0001**	
2009–2012	**0.0029**	**0.0031**		1.0000	**0.0474**	0.1958	**0.0007**	**<0.0001**	
2009–2013	0.9999	**<0.0001**		1.0000	0.9982	1.0000	**<0.0001**	**<0.0001**	
2009–2014	0.9993	0.1336		1.0000	1.0000	0.9993	**<0.0001**	0.1273	
2009–2015	1.0000	**<0.0001**		**0.0381**	1.0000	0.3014	**<0.0001**	**<0.0001**	
2010–2011	0.5277	**0.0014**		1.0000	0.9682	1.0000	**<0.0001**	**0.0004**	
2010–2012	0.9827	0.1310		1.0000	0.9716	**0.0042**	0.4302	0.5928	
2010–2013	**0.0250**	**0.0013**		1.0000	1.0000	1.0000	**<0.0001**	0.0758	
2010–2014	**0.0130**	0.8512		1.0000	0.9704	0.7615	**0.0009**	1.0000	
2010–2015	0.7893	**0.0011**		**0.0163**	0.7084	0.9735	**0.0003**	0.2270	
2011–2012	**0.0091**	0.9957		1.0000	0.1419	**0.0005**	**0.0250**	0.5077	
2011–2013	0.9954	1.0000		1.0000	1.0000	0.9999	0.9886	0.9977	
2011–2014	0.9866	0.7164		0.9999	1.0000	0.4674	0.9796	**0.0050**	
2011–2015	1.0000	1.0000		**0.0076**	1.0000	1.0000	0.7658	0.8097	
2012–2013	**<0.0001**	0.9728		1.0000	0.5050	**0.0070**	**<0.0001**	0.9967	
2012–2014	**<0.0001**	0.9986		1.0000	0.1228	0.8572	0.7638	0.6908	
2012–2015	**0.0361**	0.9997		**0.0079**	**0.0093**	**<0.0001**	0.8612	1.0000	
2013–2014	1.0000	0.2713		1.0000	0.9999	0.8392	**0.0376**	**0.0279**	
2013–2015	0.9448	1.0000		0.0441	0.9927	0.8415	**0.0035**	1.0000	
2014–2015	0.8285	0.5484		0.0473	1.0000	**0.0029**	1.0000	0.1759	

Significant multiple comparisons (α = 0.05; p ≤ 0.05) are highlighted in bold. Comparison = the years compared and T6 = Total 6-prey group biomass. TS corr. = time series correlation model, AR(2) = autoregressive model with 2 lags, AR(3) = autoregressive model with 3 lags, MA(2) = moving average with 2 lags, and ARMA = autoregressive and moving average model, both with 2 lags.

**Table 5:** Tukey multiple comparisons among years for faunal groups for the offshore surveys, 2002–2015.

Comparison	Amphipoda	Bivalvia	Cumacea	Polychaeta	T6
Model	D + Y	Y	D + Y	D + Y	D + Y
TS Corr.	ARMA(3,3)	ARMA(2,2)	ARMA(2,2)	ARMA(2,2)	ARMA(2,2)
2002–2003	1.0000	**0.0413**	0.3923	0.1087	0.6956
2002–2004	1.0000	**0.0002**	0.7877	0.9867	0.9594
2002–2005	1.0000	0.0180	0.2087	0.9306	0.8997
2002–2006	1.0000	0.9990	1.0000	0.9298	1.0000
2002–2007	**0.0044**	0.9953	0.9674	1.0000	0.3045
2002–2008	0.9008	0.9338	0.0555	0.9043	0.2449
2002–2009	0.1129	0.1624	0.9183	0.9996	1.0000
2002–2010	0.9878	0.0700	**0.0011**	1.0000	0.9993
2002–2012	**<0.0001**	**0.0001**	**0.0002**	0.2527	0.9992
2002–2013	**<0.0001**	**<0.0001**	0.3851	**0.0243**	0.9916
2002–2014	**0.0002**	**<0.0001**	0.1863	**0.0446**	0.9977
2002–2015	**0.0003**	**<0.0001**	0.3389	**0.0121**	0.7826
2003–2004	1.0000	0.8730	1.0000	0.9737	1.0000
2003–2005	1.0000	1.0000	1.0000	0.9815	1.0000
2003–2006	1.0000	0.5117	0.2357	0.9895	0.7972
2003–2007	**0.0043**	**0.0008**	0.9999	**0.0314**	**0.0005**
2003–2008	0.9482	**0.0001**	**<0.0001**	**0.0005**	**0.0003**
2003–2009	0.1498	1.0000	1.0000	**0.0125**	0.7993
2003–2010	0.9968	1.0000	0.5488	0.5052	0.9997
2003–2012	**<0.0001**	0.9123	0.2664	1.0000	0.2083
2003–2013	**<0.0001**	**0.0065**	1.0000	0.9999	1.0000
2003–2014	**0.0003**	0.3810	1.0000	1.0000	1.0000
2003–2015	**0.0005**	**0.0019**	1.0000	0.9992	1.0000
2004–2005	1.0000	0.9414	0.9999	1.0000	1.0000
2004–2006	1.0000	**0.0033**	0.3338	1.0000	0.9158
2004–2007	**0.0099**	**<0.0001**	1.0000	0.7498	**0.0020**
2004–2008	0.9867	**<0.0001**	**<0.0001**	0.1201	**0.0015**
2004–2009	0.2530	0.7377	1.0000	0.5613	0.9196
2004–2010	0.9997	0.9056	0.5002	0.9997	1.0000
2004–2012	**<0.0001**	1.0000	0.2401	0.9874	0.3746
2004–2013	**<0.0001**	0.6861	1.0000	0.6652	1.0000
2004–2014	**0.0010**	1.0000	0.9998	0.7816	1.0000

Comparison	Amphipoda	Bivalvia	Cumacea	Polychaeta	T6
2004–2015	**0.0014**	0.4676	1.0000	0.5082	1.0000
2005–2006	1.0000	0.0894	**0.0021**	1.0000	0.6058
2005–2007	**0.0002**	**<0.0001**	0.9592	0.4042	**0.0001**
2005–2008	0.9197	**<0.0001**	**<0.0001**	**0.0204**	**0.0001**
2005–2009	0.0748	1.0000	0.9940	0.2594	0.8002
2005–2010	0.9972	1.0000	0.8307	0.9951	0.9999
2005–2012	**<0.0001**	0.9692	0.5280	0.9903	0.1811
2005–2013	**<0.0001**	**0.0076**	1.0000	0.6239	1.0000
2005–2014	**0.0001**	0.4933	1.0000	0.7603	1.0000
2005–2015	**0.0002**	**0.0021**	1.0000	0.4540	1.0000
2006–2007	**<0.0001**	0.3203	0.3780	0.2298	**0.0349**
2006–2008	0.9125	0.1501	**0.0421**	**0.0135**	0.0828
2006–2009	0.0600	0.6124	0.4948	0.2288	1.0000
2006–2010	0.9984	0.3892	**<0.0001**	0.9938	0.9980
2006–2012	**<0.0001**	**0.0019**	**<0.0001**	0.9920	0.9982
2006–2013	**<0.0001**	**<0.0001**	0.0659	0.6449	0.9798
2006–2014	**0.0001**	**<0.0001**	**0.0188**	0.7782	0.9939
2006–2015	**0.0002**	**<0.0001**	0.0543	0.4768	0.6488
2007–2008	0.0554	1.0000	**<0.0001**	0.9807	1.0000
2007–2009	0.9985	**0.0003**	1.0000	1.0000	0.1458
2007–2010	0.0925	**0.0001**	0.0548	0.9920	**0.0052**
2007–2012	**0.0495**	**<0.0001**	**0.0138**	**0.0188**	0.8439
2007–2013	0.4141	**<0.0001**	0.9948	**0.0005**	**0.0019**
2007–2014	0.9859	**<0.0001**	0.9426	**0.0012**	**0.0041**
2007–2015	0.9914	**<0.0001**	0.9889	**0.0002**	**0.0001**
2008–2009	0.6368	**<0.0001**	**<0.0001**	0.9990	**0.0280**
2008–2010	1.0000	**<0.0001**	**<0.0001**	0.3915	**0.0015**
2008–2012	**<0.0001**	**<0.0001**	**<0.0001**	0.0001	0.7550
2008–2013	**<0.0001**	**<0.0001**	**<0.0001**	**<0.0001**	**0.0010**
2008–2014	**0.0109**	**<0.0001**	**<0.0001**	**<0.0001**	**0.0024**
2008–2015	**0.0198**	**<0.0001**	**<0.0001**	**<0.0001**	**<0.0001**
2009–2010	0.2747	1.0000	**0.0178**	0.8717	0.9811
2009–2012	**0.0001**	0.5881	**0.0151**	**0.0031**	0.9984
2009–2013	**0.0176**	**0.0003**	0.9992	**0.0001**	0.9648
2009–2014	0.5311	0.1166	0.9819	**0.0003**	0.9891
2009–2015	0.6324	**0.0001**	0.9980	**<0.0001**	0.5882
2010–2012	**<0.0001**	0.7537	1.0000	0.2194	0.3635
2010–2013	**<0.0001**	**0.0007**	0.5934	**0.0329**	1.0000
2010–2014	**0.0003**	0.2393	0.8969	0.0794	1.0000
2010–2015	**0.0014**	**0.0004**	0.7858	**0.0234**	0.9969
2012–2013	0.9987	0.2182	0.1115	0.9981	0.1617
2012–2014	0.5788	0.9991	0.5554	0.9999	0.4733

Comparison	Amphipoda	Bivalvia	Cumacea	Polychaeta	T6
2012–2015	0.6891	0.1756	0.4501	0.9948	0.0663
2013–2014	0.9595	0.8804	1.0000	1.0000	1.0000
2013–2015	0.9803	1.0000	1.0000	1.0000	0.9998
2014–2015	1.0000	0.6517	1.0000	1.0000	0.9952

Significant multiple comparisons (α = 0.05; p ≤ 0.05) are highlighted in bold. Comparison = the years compared and T6 = Total 6-prey group biomass. Model = the regression model, D = water depth, Y = year, TS corr. = time series correlation model, AR(2) = autoregressive model with 2 lags, ARMA(2,2) = autoregressive and moving average model, both with 2 lags, and. ARMA(3,3) = autoregressive and moving average model, both with 3 lags.

**Table 6:** Analysis of variance of nine prey groups from feeding points in the nearshore and offshore study areas, 2002–2015.

Taxon	Piltun			Taxon	Offshore		
	Est.	P-Value	Comparison		Est.	P-Value	Comparison
Amphipoda	0.91	<0.0001	FP>GS	Amphipoda	0.84	<0.0001	FP>GS
Bivalvia	0.13	0.1891		Bivalvia	−0.20	0.6907	
Cumacea	0.14	0.9340		Cumacea	−0.46	<0.0001	GS>FP
Isopoda	0.67	<0.0001	FP>GS	Polychaeta	0.08	0.2404	
Actinopterygii	−0.02	0.8683		Total Prey	0.44	<0.0001	FP>GS
Polychaeta	0.02	0.7072					
Total Prey	0.56	<0.0001	FP>GS				

Est. = the difference between feeding point biomass – grid station biomass for transformed biomass data used in the mixed models, and P-values from mixed models. A positive estimate value indicates that average biomass was higher at feeding points. The “Comparison” columns denote whether biomass was higher in feeding points (FP) or grid stations (GS). Years included in the ANOVA were 2002–2012 for the nearshore and 2002–2015 for the offshore.

**Table 7:** Correlations among environmental variables canonical correspondence analysis axes, cumulative proportion of variance accounted for, and permutational analysis of variance for variables and axes (p-values) for the Piltun and Offshore study areas, 2002–2015. Correlations ≥ |0.30| are in bold.

Piltun	CCA1	CCA2	CCA3	CCA4	P-Values
Sand	−0.18	−0.08	−0.01	−0.09	0.001
Year	−0.07	**−0.47**	−0.01	0.01	0.001
Depth	**0.67**	−0.01	0.01	0.00	0.001
ALPI	−0.01	0.09	0.14	0.00	0.079
P-Values	0.001	0.001	0.341	0.944	
Cum Prop. Var.	9%	11%	12%	12%	
**Offshore**	CCA1	CCA2	CCA3	CCA4	P-Values
Sand	0.22	−0.12	0.17	−0.02	0.001
Year	**0.30**	−0.15	−0.12	−0.01	0.002
Depth	**−0.49**	−0.09	−0.07	−0.02	0.001
ALPI	−0.02	0.11	−0.01	−0.07	0.337
P-Values	0.001	0.040	0.483	0.990	
Cum Prop. Var.	6%	7%	8%	8%

**Table 8:** Analysis of covariance for the nearshore and offshore study areas adjacent to Sakhalin Island, 2001–2015.

Nearshore				Offshore		
Group	Factor	F	P-value	Factor	F	P-value
Actinopterygii	Depth	0.1	0.7782	Depth	7.8	0.0053
	Year	8.7	<0.0001	Year	7.3	<0.0001
Amphipoda	Depth	151.2	<0.0001	Depth	15.3	<0.0001
	Year	19.8	<0.0001	Year	10.4	<0.0001
Bivalvia	Depth	1.5	0.2219	Depth	0.0	0.9674
	Year	17.0	<0.0001	Year	15.5	<0.0001
Cumacea	Depth	15.3	<0.0001	Depth	63.3	<0.0001
	Year	39.2	<0.0001	Year	11.1	<0.0001
Isopoda	Depth	84.3	<0.0001	Depth	12.2	<0.0001
	Year	10.2	<0.0001	Year	3.2	<0.0001
Polychaeta	Depth	10.0	<0.0001	Depth	63.2	<0.0001
	Year	18.5	<0.0001	Year	6.0	<0.0001
Total Prey	Depth	87.1	<0.0001	Depth	3.8	0.0515
	Year	13.4	<0.0001	Year	5.9	<0.0001

Mixed models were adjusted for time-series errors. F-statistics (F) and p-values are presented.

## 2 Experimental design, materials, and methods

### 2.1 Sampling

Bottom sampling was conducted in the summers of 2001–2015 to measure biomass concentrations and environmental characteristics in the GW feeding area adjacent to northeastern Sakhalin Island (the Sakhalin feeding area; Fig. 1). Sampling was conducted aboard the *R/V Okean* (2001), *R/V Nevelskoy* (2002–2003), *R/V Academic Oparin (2*004–2010), *R/V Academic Lavrentiev* (2005), and *R/V Igor Maksimov* (2011–2015). Benthic biological and sediment samples were collected using a van Veen grab with a surface area of 0.2 m^2^. Research vessel drafts limited operations of van Veen grabs to water depths ≥9 m though some slightly shallower samples (7–8 m) were occasionally collected. Onboard, samples were sieved over a 1.0-mm-mesh screen and the organisms preserved in 4% formalin. In the taxonomic laboratory, biological material was sorted from the sediment residues and animals were identified, counted, and weighed. Animals were grouped into classes or higher taxonomic categories. Sediment grain-size analyses were used to determine standard grain-size categories.

Initial investigation of the nearshore feeding area was conducted in 2001 by divers to explore prey habitat (5–30 m water depth) and provide a basis for designing the nearshore Piltun survey grid. The current nearshore sampling grid consists of 72 cells along the northeastern coast of Sakhalin Island encompassing the nearshore (<20 m) GW feeding habitat and extending to deeper waters to capture environmental gradients. The total area of the nearshore survey area is approximately 1100 km^2^. Sampling was initiated in the offshore feeding area in 2002 and the offshore survey currently includes 48 cells with a total area of approximately 2000 km^2^. Data records for diver sampling in 2001 consists of single biomass estimates for sampling location. Benthos and sediment sample collections from 2002 to 2015 comprised three replicates collected at randomly selected sampling points or by repeated sampling of locations selected in previous years. Samples from 2002 to 2015 were collected in water depths ranging from 7 to 35 m nearshore and from 30 to 63 m offshore. Both survey grids in the Sakhalin Island coastal study area are adjacent to or overlap with areas of heightened anthropogenic activities including commercial fishing and oil and gas platforms (Fig. 1). During the course of the investigation, locations where gray whales were observed feeding were opportunistically sampled and three replicates collected at each point. These feeding points were identified by gray whale observers from shore and on vessels associated with oil and gas exploration and production activities. The feeding points provide further information characterizing specific locations where whales feed. Feeding areas were sampled differently in 2015 using a targeted sampling approach with six replicates collected along two transects at 9 m and 13 m for a total of twelve replicates at each location. Feeding areas from 2015 were not statistically-evaluated for differences here but were considered separately (Blanchard et al., unpublished data).

Environmental variables included water depth, year of sampling, percent sand (sand: particles between 0.1 mm and 1.0 mm; other categories were colinear with sand), and the Aleutian Low Pressure Index (ALPI). The Aleutian Low [2] influences winter wind patterns and sea-level pressure throughout the Bering Sea and variations in its strength and position can directly influence water circulation [3], [4]. The ALPI is available at https://open.canada.ca/data/.

### 2.2 Statistical analyses

Analysis of covariance was performed for surveys using mixed modeling to test for differences among years. ANCOVA's were performed separately for the nearshore (incorporating data from 2001 to 2015) and offshore surveys (using data from 2002 to 2015). The mixed-modeling package nlme [5] was used with the statistical program R [6] for analysis of as it allows incorporation of models for temporally-correlated errors. Autoregressive and moving average correlation models were used in nlme to correct for temporal correlations in yearly averages. Correcting for temporal correlations among errors increases the precision of statistical tests by correcting variances. Here, we limited our consideration to models of at most 3 lags, or up to 3 years distant. We also presumed that any spatial correlation structures would be approximated by and incorporated in the correlation models. Models considered for adjusting errors were autoregressive (AR), moving average (MA), and combined models (ARMA).

Model selection included determination of the variables appropriate for inclusion as well as the best correlation model. The available models included depth, year, and Depth and Year. Depth was a continuous variable and Year a fixed factor. Station was included as a random factor in mixed models. Akaike's Information Criterion (AIC) was used to determine the best model of the three for each faunal group analyzed. The choice for which correlation model to use was guided by likelihood ratio tests that compare the variance reductions among correlation models. Tukey multiple comparisons were performed using the lmerTestpackage in R [7].

### 2.3 Multivariate analyses

Multivariate analyses were applied to characterize changes in benthic community biomass concentrations related to environmental predictors. Canonical correspondence analysis (CCA) was used to test the hypothesis that environmental and temporal covariates were predictors of biomass community structure. The community data set was benthic biomass of all categories identified with rare animals excluded. Biomass data were *ln*(X+1)-transformed prior to analyses to reduce influences of extreme values on the ordination. The covariates were water depth, year, the ALPI (a measure of macro-scale climate variability), and percent sand. CCA was conducted using the vegan package [8].
